# Sports-Related Genomic Predictors Are Associated with Athlete Status in Chinese Sprint/Power Athletes

**DOI:** 10.3390/genes15101251

**Published:** 2024-09-26

**Authors:** Yaqi Wang, Zihong He, Tao Mei, Xiaolin Yang, Zhuangzhuang Gu, Zhihao Zhang, Yanchun Li

**Affiliations:** 1 Department of Exercise Biochemistry, Exercise Science School, Beijing Sport University, Beijing 100084, China; wangyaqi@bsu.edu.cn (Y.W.);; 2 Exercise Biology Research Center, China Institute of Sport Science, Beijing 100084, China; 3 China Institute of Sport and Health Science, Beijing Sport University, Beijing 100084, China; 4 Institute of Physical Education, Henan Normal University, Xinxiang 453007, China; 5 Beijing Key Laboratory of Sports Performance and Skill Assessment, Beijing 100084, China; 6 Key Laboratory for Performance Training & Recovery of General Administration of Sport, Beijing 100084, China

**Keywords:** athlete status, sprint/power, polymorphism, total genetic score

## Abstract

**Objectives:** The aim of this study was to assess the relationship between variant loci significantly associated with sports-related traits in the GWAS Catalog database and sprint/power athlete status, as well as to explore the polygenic profile of elite athletes. **Methods:** Next-generation sequencing and microarray technology were used to genotype samples from 211 elite athletes who had achieved success in national or international competitions in power-based sports and from 522 non-athletes, who were healthy university students with no history of professional sports training. Variant loci collected from databases were extracted after imputation. Subsequently, 80% of the samples were randomly selected as the training set, and the remaining 20% as the validation set. **Results:** Association analysis of variant loci was conducted in the training set, and individual Total Genotype Score (TGS) were calculated using genotype dosage and lnOR, followed by the establishment of a logistic model, with predictive performance evaluated in the validation set. Association analysis was performed on 2075 variant loci, and after removing linked loci (r^2^ > 0.2), 118 Tag SNPs (*p* ≤ 0.05) were identified. A logistic model built using 30 Tag SNPs (*p* ≤ 0.01) showed better performance in the validation set (AUC = 0.707). **Conclusions:** Our study identified 30 new genetic molecular markers and demonstrated that elite sprint/power athlete status is polygenic.

## 1. Introduction

Compared to non-athletes or endurance athletes, sprint/power athletes demonstrate distinct physiological characteristics, including skeletal muscle hypertrophy, a high proportion of fast-twitch muscle fibers, efficient neural conduction, a robust anaerobic energy system, and elevated serum testosterone levels [1,2,3,4]. These differences primarily result from a combination of environmental factors (e.g., training, nutrition) and genetic factors (e.g., single-nucleotide polymorphisms, DNA methylation) [5]. Genetic factors have been estimated to contribute to 66% of athletic status heritability [6]. Physiological characteristics related to athletic performance exhibit high heritability, with genetic factors accounting for 30% to 82% of the variation in these traits [7,8,9,10].

Genetic research in sports often utilizes candidate gene methods to identify genetic variations associated with athletic performance. By identifying genes influencing muscle function, energy metabolism, nervous system development, serum testosterone levels, and other physiological indicators, and analyzing genotype distribution at variant sites in gene functional regions among elite athletes and non-athletes, candidate gene variants for athlete selection are identified. A recent review indicated that 45 genetic markers related to power and 42 related to strength were positively associated with athlete status in at least two studies [11]. However, achieving elite athlete status is a complex phenomenon [12]. Candidate gene variations focus solely on a few presumed variant sites, potentially overlooking the genome’s overall complexity and gene interactions, thereby limiting their effectiveness in predicting athlete status. Some studies have sought to improve the predictive power of candidate genes by aggregating existing markers into a TGS; however, the accuracy of predictions remains constrained [13].

Genome-wide association studies (GWASs) are effective methods for identifying genetic variations associated with complex phenotypes. In human complex trait research, GWAS results demonstrate superior reproducibility [14]. However, GWASs require large sample sizes to achieve sufficient statistical power, posing a significant limitation for conducting such studies on elite athletes. To address this challenge, Semenova et al. [11] proposed using variants significantly associated with sport-related traits (*p* ≤ 5 × 10^−8^) identified in non-athlete populations and assessing them in elite athlete and non-athlete control groups. A TGS is calculated by summing the scores of favorable genes to assess genetic susceptibility to complex phenotypes. In sports genetics research, TGS is commonly used to predict the potential for endurance or power performance [15]. Recently, a study validated 70 genetic markers highly associated with a brisk walking pace in UK Biobank participants among Russian elite sprinters. It identified 15 genetic markers that exhibited significant differences between elite sprinters and controls, with the calculated TGS demonstrating strong predictive performance [16].

While previous studies have focused on polygenic profiles in European populations, research on genetic markers associated with sprint/power athletes in the Chinese population remains limited. This study aims to address this gap by utilizing the GWAS Catalog to identify and validate genetic variants associated with sports-related traits in Chinese sprint/power athletes. These genetic markers will be evaluated in both athletes and a control group of non-athletes, and a TGS will be calculated to assess its effectiveness in predicting athletic status and understanding the polygenic profile of Chinese athletes. We hypothesize that the identified genetic variants will be successfully validated and provide insights into the genetic basis of elite athletic performance.

## 2. Materials and Methods

### 2.1. Study Participants and Ethical Approval

This study included 733 Chinese participants, comprising 211 sprint/power athletes (mean height: 172.0 ± 8.3 cm, weight: 63.7 ± 9.9 kg, age: 20 ± 4 years; 94 males and 117 females) and 522 healthy controls (mean height: 176.8 ± 5.8 cm, weight: 71.3 ± 12.4 kg, age: 20 ± 3 years; 257 males and 265 females). The athletes were involved in the following disciplines: weightlifting (*n* = 126), throwing (*n* = 38), sprinting (*n* = 27), hurdling (*n* = 3), and jumping (*n* = 17). Of these, 53 athletes were classified as ‘elite’, having competed in international events and ranked among the top 10 worldwide, while 158 were classified as ‘sub-elite’, having participated in national competitions and ranked in the top 10 nationally. The control group consisted of healthy university students with no history of professional sports training.

The inclusion criteria for athletes required them to meet the definitions of ‘elite’ or ‘sub-elite’ based on their competitive level and ranking. The control group consisted of healthy individuals with no history of professional sports participation. Exclusion criteria for both groups included a history of chronic disease or injury that could affect performance, as well as any record of using prohibited stimulants. All participants provided written informed consent before their participation in this study. The study protocol was approved by the Sports Science Experiment Ethics Committee of Beijing Sport University (approval number: 2024274H) and adhered strictly to the principles of the Declaration of Helsinki.

### 2.2. Genotyping

Five milliliters of venous blood was collected from each individual into sterile tubes containing EDTA. The samples were stored at 4 °C and transported to the laboratory on the same day for DNA extraction. DNA was extracted and purified from blood leukocyte samples using the TIANGEN DNA extraction kit (Beijing, China). Whole-genome sequencing of athletes’ DNA samples was conducted using the Illumina NovaSeq 6000 platform (Illumina, San Diego, CA, USA), generating 150 bp paired-end reads at 30X coverage. The short reads obtained from sequencing were aligned to the reference genome (GRCh38) using the BWA-MEM Version 0.7.17 algorithm. Sorting and marking of PCR duplicates were performed using the MarkDuplicates module of Bcftools Version 1.10.2 and the Genome Analysis Toolkit (GATK Version 4.2.4.0). Base recalibration and genotype calling were carried out using GATK’s (Version 4.2.4.0) BaseRecalibrator module and HaplotypeCaller module, followed by variant quality filtering with the Variant Quality Score Recalibration module. Control samples were genotyped using the Illumina Chinese Genotyping Array, and GRCh37 coordinates in the microarray data were converted to GRCh38 using CrossMap Version 0.6.5.

### 2.3. Pre-Processing of Data

Using the 1000 Genomes Phase 3 data as a reference panel, pre-phasing and genotype imputation were conducted using Shapeit5 Version 5.1.1 and Minimac4 Version 1.0.2. Variants with low certainty (info score < 0.6) were filtered out post-imputation.

Of the 211 athletes, 169 (80%) were randomly assigned to the training set, and the remaining 42 (20%) to the test set. Similarly, the control group was divided with 418 (80%) in the training set and 104 (20%) in the test set.

The training set was utilized for association analysis of candidate variants and assessing the effects of TGS, whereas the test set was employed to evaluate the predictive performance of TGS. Data from the training set, comprising 169 athletes and 418 controls, were merged using Bcftools to create a unified training set of 587 samples. Data quality control was conducted using Plink Version 1.9, excluding variants with a missing rate > 0.02, deviations from Hardy–Weinberg equilibrium (*p* < 0.05), and minor allele frequencies < 0.05. Participants with a missing rate > 0.02, relatedness coefficient > 0.125, deviating heterozygosity/genotype calls (±3 standard deviations), and discrepancies in inferred versus self-reported sex were excluded.

The GWAS Catalog “https://www.ebi.ac.uk/gwas/ (accessed on 24 July 2024)” is an online resource that aggregates and curates findings from GWASs on human traits and diseases. Candidate variants were identified by querying GWASs in non-athlete populations showing strong associations (*p* ≤ 5 × 10^−8^) with traits related to explosive sports performance, including physical activity, brisk walking, hand grip strength, lower body strength, reaction time, lean body mass, muscle mass, sarcopenia, IGF-1 levels, cortisol levels, myoglobin levels, testosterone levels, and interleukin-6 levels. Hand grip strength and lower body strength are direct indicators of muscular power, which is essential for explosive movements in sprinting and power sports. Physical activity and brisk walking serve as indicators of general fitness, reflecting an individual’s overall exercise adaptability. Reaction time is crucial for explosive sports performance, as faster neural responses allow athletes to swiftly execute powerful movements in response to stimuli. Lean body mass and muscle mass enhance force production, which is a key factor in speed and strength-based activities. Sarcopenia, the loss of muscle mass, impairs performance by reducing strength and explosiveness. IGF-1 and testosterone are vital for muscle growth, recovery, and overall athletic performance, particularly in high-intensity sports. Cortisol and interleukin-6 regulate stress responses and inflammation, impacting recovery and resilience during intense training. Myoglobin supports oxygen transport in muscles, playing a crucial role in skeletal muscle recovery following high-intensity training. These variants were extracted from the training set for subsequent analysis.

### 2.4. Statistical Analyses

Univariate logistic regression analyses were conducted on candidate variants in the training set using PLINK 1.9 to compute odds ratios (ORs) and associated *p*-values, comparing variants between athletes and controls.

Using the Clumping and Thresholding (C+T) method to filter variants within the same linkage disequilibrium (LD) region, we applied a significance threshold (*p* ≤ 0.01 or *p* ≤ 0.05) to identify Tag SNPs. Variants with *p*-values higher than that of the index variant and with high linkage (r² > 0.2) to the index variant were excluded. Only variants located within 250 kb of the physical position of the index variant were included. TGS was calculated using the following formula:

$${TGS}_{i}=\frac{\sum_{i=1}^{n}S_{i}\times G_{ij}}{P\times M_{j}}$$

For each genetic variant (i) in an individual (j), the product of the effect size ($S_{i}$) and the genotype dosage ($G_{ij}$) were calculated. These products were summed across all variants filtered by the C+T method to obtain the total score. The TGS was standardized by dividing the sum by the product of the ploidy (*P*) of an individual (2 for humans) and the number of non-missing variants in the sample ($M_{j}$). The effect size was represented as the natural logarithm of the odds ratio (lnOR). The genotype dosage was assigned as follows: 0 for wild type, 1 for heterozygous mutant, and 2 for homozygous mutant.

In the training set, the classification outcome (athletes or controls) was used as the dependent variable (*y*) and the individual’s TGS (${TGS}_{i}$) as the independent variable (*x*). The R package caret was employed to perform five-fold cross-validation and build a univariate logistic regression model. The model’s predictive ability was evaluated by calculating the area under the curve (AUC).

## 3. Results

### 3.1. Association Analysis

In the GWAS Catalog database, we initially identified 7773 variants significantly associated (*p* ≤ 5 × 10^−8^) with various traits: 2777 for testosterone measurement, 1608 for lean body mass, 514 for IGF-1 measurement, 230 for hand grip strength, 167 for reaction time measurement, 75 for brisk walking, 69 for interleukin-6 measurement, 62 for muscle measurement, 20 for thigh muscle volume, 14 for cortisol measurement, 3 for myoglobin measurement, 3 for lower body strength measurement, and 1 for sarcopenia (Supplementary Table S1).

**Figure 1:**
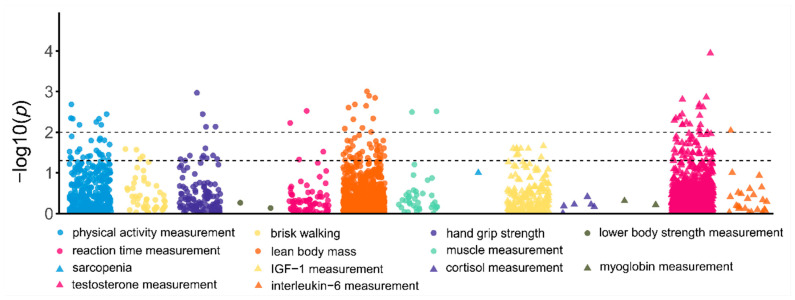
Genetic markers strongly associated with sports-related traits from the GWAS Catalog and their association with athlete status in Chinese sprint/power athletes. Each data point on the graph represents a genetic variant, with the log10-transformed *p*-value of its association displayed on the *y*-axis. The *p*-values were derived by assessing the association between the genetic variants and sprint/power athlete status in comparison to non-athletes. The traits indicated in the legend correspond to phenotypes identified in the GWAS Catalog and were not directly measured in our athlete or control samples. The dashed lines indicate significance thresholds (*p* ≤ 0.01 or *p* ≤ 0.05).

After removing duplicate genetic variants and conducting quality control on whole-genome sequencing data, a total of 2075 variant loci were included for further association analysis. Logistic regression performed using PLINK in the training set identified 164 variants with *p*-values ≤ 0.05, 44 variants with *p*-values ≤ 0.01, and no variants meeting the Bonferroni-corrected threshold (*p* ≤ 0.05/2075) (Figure 1). Utilizing the C+T method to account for LD, we isolated 118 Tag SNPs with *p*-values ≤ 0.05 and 30 Tag SNPs with *p*-values ≤ 0.01 (Supplementary Table S2).

### 3.2. Polygenic Analysis

**Figure 2:**
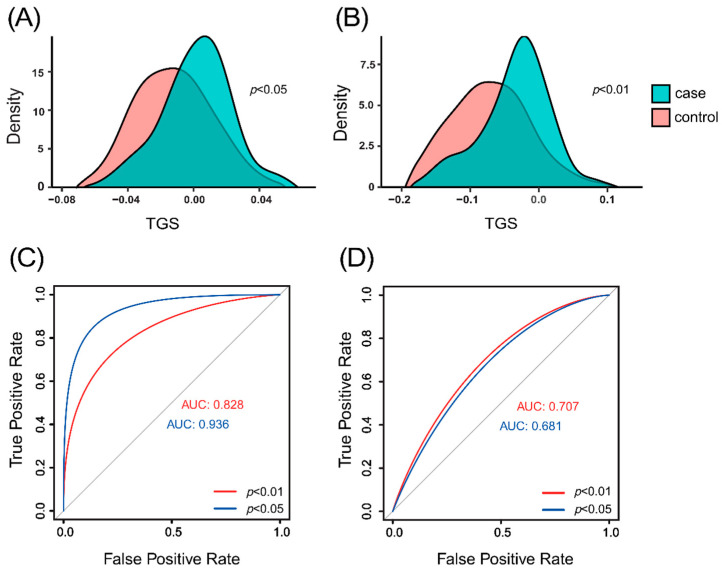
Standardized distribution of TGS and predictive performance of logistic regression models in athletes and controls. (**A**) Standardized distribution of TGS calculated in the validation set after filtering with *p* ≤ 0.05. (**B**) Standardized distribution of TGS calculated in the validation set after filtering with *p* ≤ 0.01. (**C**) Predictive performance of the logistic regression model in the training set. (**D**) Predictive performance of the logistic regression model in the validation set. Abbreviations: AUC = area under the curve, TGS = Total Genotype Score, *p* = *p*-value.

Tag SNPs were filtered using two thresholds (*p* ≤ 0.01 or *p* ≤ 0.05) to calculate TGS and assess logistic regression models in both the training and validation sets. Figure 2A displays the standardized distribution of TGS calculated from Tag SNPs filtered with *p* ≤ 0.05 in the validation set, while Figure 2B illustrates the standardized distribution of TGS from Tag SNPs filtered with *p* ≤ 0.01 in the validation set. Logistic regression models utilizing Tag SNPs filtered with *p* ≤ 0.05 achieved anAUC of 0.936 in the training set and 0.681 in the validation set. For Tag SNPs filtered with *p* ≤ 0.01, the AUCs were 0.828 in the training set and 0.707 in the validation set (Figure 2C,D).

TGS calculated using the *p* ≤ 0.05 threshold shows overfitting in predicting athletic status and lower predictive capability in the validation set compared to TGS calculated with the *p* ≤ 0.01 threshold. Table 1 presents the association and annotation results for 30 Tag SNPs, comprising 29 SNPs and 1 INDEL, all located in non-coding gene regions. Among these, 11 Tag SNPs are from GWASs on testosterone measurement, 2 from reaction time measurement, 3 from physical activity measurement, 10 from lean body mass, 1 from interleukin-6 measurement, and 3 from hand grip strength.

## 4. Discussion

This study validates genetic variant loci retrieved from the GWAS Catalog database in both athletes and controls, identifying 30 candidate gene loci potentially linked to the athlete status of Chinese sprint/power athletes.

Sprint/power athletic status is a complex trait influenced by interactions among multiple genes. Therefore, traditional candidate gene association results are often limited in their ability to replicate results and identify talent [17]. Similarly, the predictive performance of TGS calculated using known candidate genetic variants remains limited in validation cohorts. Homma et al. [13] developed a TGS model using six candidate genes associated with weightlifter status. They found that while the average TGS was significantly higher in elite weightlifters compared to controls, no significant differences were observed in wrestlers, powerlifters, and throwers compared to controls, indicating limited generalizability of TGS models based on candidate genes. Guilherme et al. [18] calculated TGS using 2 to 10 SNPs between 378 Brazilian athletes and 818 controls. They observed that using a greater number of polymorphic loci for scoring resulted in stronger differences and improved discriminatory ability. When using TGS calculated with 10 relevant polymorphic loci, it effectively differentiated athlete groups, but its predictive accuracy remained limited (AUC = 0.61). Miyamoto-Mikami et al. [19] calculated TGS using 21 candidate gene markers in Japanese sprint/power track and field athletes. Their results revealed no significant differences in average TGS between athletes and controls, suggesting that existing candidate gene markers may not be applicable to East Asian populations.

Our study suggests that utilizing GWAS results of sports-related traits for associations in athletes and applying more stringent thresholds (*p* ≤ 0.01) to filter Tag SNPs using lnOR as the effect size results in a TGS that exhibits enhanced predictive performance in the validation dataset (AUC = 0.707). This indicates that athletic status shares genetic characteristics with complex phenotypes, where numerous variant loci across the genome influence the phenotype, often distributed widely in non-coding regions rather than the expected coding regions of candidate genes [14]. Previous studies on athletic TGS typically used genotype dosage or the count of individuals carrying the dominant genotype as measures, giving equal weight to all SNPs in the total score and disregarding variations in the effects of different genes [18], employing lnOR as the effect size enables the differentiation of contributions from various genes, thereby enhancing the TGS’s capability to differentiate between sprint/power athletic and nonathletic individuals. Although our TGS shows improved predictive performance compared to previous studies, its accuracy remains moderate. Additionally, our aim in calculating athlete TGS is to uncover the multigenic characteristics of elite athletes rather than assigning genetic scores to individual athletes.

Thirty variant loci were annotated to forty genes, including ten variant loci located between genes. These genes are primarily associated with neurodevelopment (*DNMBP*, *ATXN2L*, *GAD1*, *GPR139*, *CLN3*, *FOXP1*, *H4C8*, *TFAP2D*, *TFAP2B*), skeletal muscle function (*MBNL1*, *MAP2K5*), mitochondrial function (*FAM124B*, *MAIP1*, *ATG13*), and metabolism (*NR1D2*, *P2RY2*) (Supplementary Table S3).

Among the 230 variant loci potentially influencing hand grip strength, 3 variants are associated with sprint/power athletic status and are annotated to genes associated with neurodevelopment. The *FOXP1* gene is a crucial transcription factor that influences early brain and organ development, particularly in neurodevelopment. Mutations or deletions in this gene can lead to FOXP1 syndrome, causing symptoms like hypotonia, motor dysfunction, and visual–motor integration deficit [20,21]. *H4C8* is a protein-coding gene associated with Tessadori–Bicknell–Van Haaften neurodevelopmental syndrome 2 and Tessadori–Bicknell–Van Haaften neurodevelopmental syndrome 1. Expression levels of *NOTCH2*, *H4C8,* and *H2BC21* in the substantia nigra of Parkinson’s disease patients effectively distinguish them from controls [22]. *TFAP2D*’s specific function is currently unclear, but it has been found to correlate with athlete status in Russian professional strength athletes [1]. TFAP2B forms dimers with TFAP2A, promoting the induction of the neural plate border and the specialization of neural crest cells during embryonic development [23]. Guilherme et al. [16] found that the *CRTC1* gene is highly correlated not only with brisk walking pace (using UK Biobank participants) but also with elite Russian sprinters. The *CRTC1* gene is necessary for dendritic growth in response to neuronal activity, and the *CCT3* gene, encoding a chaperonin complex subunit, is crucial for neuron branching, synaptic growth, and neuromuscular junction development. This study also found an association between the *CRTC1* gene and sprint/power athletic status in Chinese athletes (0.01 < *p* < 0.05). Therefore, we hypothesize that genetic variations influence sprint/power athletic performance not only through genes related to skeletal muscle function (*ACTN3*, *MSTN*, etc.) but also through their impact on the nervous system.

The *MBNL1* gene demonstrates a significant correlation with testosterone levels in non-athlete populations and belongs to the MBNL protein family. MBNL is highly expressed in skeletal muscle and neuronal tissues, playing a crucial role in the final differentiation of muscle cells and neurons. MBNL1 also plays a significant role in myotonic dystrophy type 1 (DM1) muscle dysfunction. Additionally, it inhibits autophagy through the mTOR pathway, thus reversing the proliferation defects of skeletal muscle satellite cells in DM1 [24]. Similarly, the *MAP2K5* gene shows a significant correlation with physical activity in non-athlete populations. It belongs to the MAP kinase family and interacts with MAPK7/ERK5, activating the latter. ERK5 and its upstream activator MEK5 are abundant in skeletal muscle, playing a crucial role in early muscle cell differentiation [25]. Therefore, MBNL1 and MAP2K5 may influence sprint/power athletic status through their effects on skeletal muscle function.

Our study presents several limitations. Firstly, the training set includes a small sample of athletes (*n* = 169), and the validation set similarly comprises a small sample of athletes (*n* = 42), which is a common limitation in studies involving elite athletes. Secondly, despite employing a more stringent threshold (*p* ≤ 0.01) for variant screening, none of the variant sites remained significant after correction, indicating a potential risk of false positives in our results. Thirdly, the significant variant sites and TGS identified in this study have not been replicated in populations from different geographic and ethnic backgrounds, which limits the broader interpretation and generalizability of the findings. As TGS and logistic regression are relatively simple models, we speculate that they may have limited applicability in other ethnic populations. Given the intricate relationship between genetic variants and sprint/power athletic status, future research should explore advanced models and algorithms, such as machine learning and deep learning, to thoroughly investigate this complex relationship.

## 5. Conclusions

This study validated and analyzed GWAS results from the GWAS Catalog, identifying 30 genetic variants associated with sprint/power athlete status among Chinese athletes, highlighting the polygenic nature of athletic performance. These findings have potential applications in areas such as athlete selection and personalized training. By identifying genetic predispositions related to performance, coaches and sports scientists could use this information to tailor training programs and optimize athlete development. Furthermore, the genetic markers identified in this study could serve as a reference for future talent identification programs, aiding in the identification of individuals with potential in sprint/power sports.

## Figures and Tables

**Table 1:** Thirty genetic variant loci associated with power/sprinter athlete status in Chinese athletes.

rsID	Localization	Alleles	Nearest Gene	OR	*p*-Value	Traits in GWAS Catalog
rs12946520	downstream	A/G	*SPEM3*	0.49	1.13 × 10^−4^	testosterone measurement
rs1112195	intergenic	T/C	*NR1D2,LINC00691*	0.59	1.37 × 10^−3^	testosterone measurement
rs11235688	UTR3	C/T	*P2RY2*	0.61	1.55 × 10^−3^	testosterone measurement
rs12443881	intronic	T/G	*ATXN2L*	0.52	2.06 × 10^−3^	testosterone measurement
rs12261919	intronic	G/C	*DNMBP*	0.64	2.41 × 10^−3^	testosterone measurement
rs7610095	intronic	G/A	*MBNL1*	1.47	4.23 × 10^−3^	testosterone measurement
rs11705936	intronic	C/T	*CCDC12*	0.69	4.53 × 10^−3^	testosterone measurement
rs28576256	intronic	A/G	*BCL11B*	1.70	5.10 × 10^−3^	testosterone measurement
rs5752773	intronic	G/A	*CHEK2*	0.62	6.43 × 10^−3^	testosterone measurement
rs1822246	intergenic	G/T	*LINC00924,LOC105369212*	1.51	6.44 × 10^−3^	testosterone measurement
rs2583949	ncRNA_intronc	T/C	*RPSAP52*	0.53	6.62 × 10^−3^	testosterone measurement
rs4606447	intronic	G/A	*ATG13*	1.47	2.99 × 10^−3^	reaction time measurement
rs4673905	intergenic	G/A	*MAIP1,SPATS2L*	1.43	5.93 × 10^−3^	reaction time measurement
rs1465370	intergenic	T/G	*CPA5,CPA1*	0.67	4.49 × 10^−3^	physical activity measurement
rs1998710	ncRNA_intronc	C/T	*LINC01720*	0.68	4.68 × 10^−3^	physical activity measurement
rs2241423	intronic	C/T	*MAP2K5*	1.43	5.62 × 10^−3^	physical activity measurement
rs10956487	intergenic	A/G	*CCDC26,GSDMC*	0.59	9.90 × 10^−4^	lean body mass
rs16909970	intergenic	G/C	*PTCH1,LINC00476*	1.63	1.43 × 10^−3^	lean body mass
rs151181	intronic	A/G	*CLN3*	0.51	2.07 × 10^−3^	lean body mass
rs3219175	upstream	G/A	*RETN*	1.70	2.24 × 10^−3^	lean body mass
rs17010957	intronic	G/A	*ARHGAP24*	0.59	2.48 × 10^−3^	lean body mass
rs7998317	intronic	C/T	*DOCK9*	1.44	4.58 × 10^−3^	lean body mass
rs11902369	intergenic	GTT/G	*GAD1,GORASP2*	0.68	4.81 × 10^−3^	lean body mass
rs9322334	intronic	T/C	*ESR1*	0.66	7.82 × 10^−3^	lean body mass
rs4291686	intergenic	G/A	*DDX6,CXCR5*	0.69	8.16 × 10^−3^	lean body mass
rs868554	intronic	G/C	*GPR139*	0.69	9.86 × 10^−3^	lean body mass
rs6722871	intergenic	A/G	*SERPINE2,FAM124B*	0.64	9.12 × 10^−3^	interleukin-6 measurement
rs34916901	upstream	G/C	*H4C8*	1.77	1.07 × 10^−3^	hand grip strength
rs4677611	intronic	A/G	*FOXP1*	1.46	7.30 × 10^−3^	hand grip strength
rs2744475	intergenic	C/T	*TFAP2D,TFAP2B*	1.43	7.39 × 10^−3^	hand grip strength

Abbreviations: rsID = rs number; OR = odds ratio.

## Data Availability

The data presented in this study are available on request from the corresponding author. The data are not publicly available due to ethical considerations and privacy concerns.

## Supplementary Materials

The following supporting information can be downloaded at https://www.mdpi.com/article/10.3390/genes15101251/s1, Table S1: Search results for genetic variants in the GWAS Catalog; Table S2: The results of association analysis for genetic variants; Table S3: Genes containing genetic variants significantly associated with power/sprinter athlete status.
